# Partially Observable Predictor Models for Identifying Cognitive Markers

**DOI:** 10.1007/s42113-025-00238-8

**Published:** 2025-03-24

**Authors:** Zita Oravecz, Martin Sliwinski, Sharon H. Kim, Lindy Williams, Mindy J. Katz, Joachim Vandekerckhove

**Affiliations:** 1 The Pennsylvania State University, Harrisburg, PA 16802 USA; 2 University of California, Irvine, Irvine, CA 92697 USA; 3 Department of Neurology, Albert Einstein College of Medicine, New York, NY 10461 USA

## Abstract

Repeated assessments of cognitive performance yield rich data from which we can extract markers of cognitive performance. Computational cognitive process models are often fit to repeated cognitive assessments to quantify individual differences in terms of substantively meaningful cognitive markers and link them to other person-level variables. Most studies stop at this point and do not test whether these cognitive markers have utility for predicting some meaningful outcomes. Here, we demonstrate a *partially observable predictor* modeling approach that can fill this gap. Using this approach, we can simultaneously extract cognitive markers from repeated assessment data and use these together with demographic covariates for predictive modeling of a clinically interesting outcome in a Bayesian multilevel modeling framework. We describe this approach by constructing a predictive process model in which features of learning are combined with demographic variables to predict mild cognitive impairment and demonstrate it using data from the Einstein Aging Study.

## Introduction

Digital technology has enabled us to collect large volumes of cognitive performance data from an individual with relative ease. For example, the well-known Project Implicit data set now contains data obtained from 2.7 million individuals for one of the dozens of cognitive performance tests (Stier et al., 2024). In other instances, people have shown great willingness to play “brain games” on smartphones in daily life settings—that is, to complete brief cognitive assessments in their natural environment repeatedly during the day, for several days (Thompson et al., 2022). Such high-frequency performance data are generated by multiple underlying processes related to learning and variability in cognitive performance on various timescales (e.g., day-to-day, week-to-week). Computational cognitive psychometric modeling (Batchelder, 2010) is needed to disentangle these latent processes and explore individual differences therein.

Over the past decades, numerous computational cognitive process models that capture the latent processes underlying observed scores of cognitive tasks have been developed. These models define cognitive parameters—unobservable or “latent” underlying features of behavior—that can be inferred from behavioral data to understand participant performance and explain variability within and between participants. Among the more popular models are the family of drift-diffusion models (Ratcliff & McKoon, 2008; Vandekerckhove et al., 2011) that separate processing speed from metacognitive factors in reaction time tasks; the expectancy-valence model (Wetzels et al., 2010) that captures risk attitudes in decision-making; Rescorla-Wagner models (Browning et al., 2015) that quantify processes in Pavlovian learning; multinomial processing tree models that quantify abilities and biases in behaviors leading to discrete outcomes (Erdfelder et al., 2009); and retest learning models that disentangle multi-timescale processes in repeated testing (Oravecz et al., 2022). The common element among these models is that they propose concrete data-generating mechanisms underlying the observed behaviors during a cognitive task. As quantitative models, they use latent variables (i.e., cognitive parameters or markers) to capture the most important characteristics of human decision-making, learning, and memory.

Untangling the sources of individual differences in these latent cognitive features is a major focus of research in cognitive science. Cognitive markers have been linked to person-level characteristics such as age (Thapar et al., 2003), anxiety (Charpentier et al., 2016), sex (Oravecz et al., 2014), and cognitive impairment (Oravecz et al., 2025), among others. However, while these studies have collectively established the validity of various cognitive markers to describe meaningful individual differences, and yielded insights that are not accessible from simple summary statistics (Yechiam et al., 2005), they often do not test whether particular cognitive markers are individually predictive of meaningful criteria—for example, *clinical outcomes* such as a diagnosis of mild cognitive impairment (MCI) or Alzheimer’s dementia (AD).

It is common practice to evaluate how much variance in model parameters is explained by a clinically meaningful outcome—using the clinical outcome as a predictor and parameters as the criterion (e.g., Hernaus et al., 2018; Ratcliff et al., 2022).^1^ In many interesting cases, however, it is useful to use person-specific latent cognitive markers more directly for the prediction of clinically meaningful outcomes. In this paper, our inference will be directly towards predicting whether a participant has the clinical condition, given their task behavior and performance. We will demonstrate this through the application of a learning model that captures practice effects in cognitive testing and show how model-based latent cognitive markers can be combined with manifest predictors into a *partially observable predictor model*. For example, rather than capturing how much variance in person-specific learning rates is explained by participants’ clinical MCI status, we will formulate a partially observable predictor model for predicting the risk for MCI as an outcome.

## A Partially Observable Predictor Model

Here, we define the *partially observable predictor* (POP) model class. The distinguishing feature of POP models is that they use both manifest (observable) and latent (unobservable) variables in order to predict a given outcome. The latent variables are identified by participants’ behavior when completing a cognitive task and must be inferred with a generative model. The key components of the POP model are (a) a generative model for extracting latent features and (b) a structural model to combine observable and unobservable predictors into a single predictive value. We will discuss these two component models first, before reviewing the Bayesian inference procedure we use to apply POP models to data.

Note that, throughout, we use “manifest” and “latent” interchangeably with “observable” and “unobservable,” res-pectively. We use “prediction” in the statistical sense, which does not imply that the criterion happens or is observed at a later time.

### Process Model to Identify Latent Features

We start by specifying a model with which we can extract latent cognitive features from high-frequency data. For repeated measures of performance scores collected from a participant *i*, at occasions *t*, we model the data $y_{ti}$ as a function of latent cognitive markers that represent theoretically meaningful constructs. In our illustrative example, we use an exponential model of practice—a learning process model that captures practice effects in repeated cognitive testing (Heathcote et al., 2000). However, we emphasize that this process model could take the form of any other parametric model that proposes latent data-generating mechanisms of a set of observations—the model could be simple (e.g., Gaussian distributions with some mean and standard deviation), but here, we introduce a process model with interpretable parameters.

Our selected process model of practice effects, the exponential learning model, is specified as follows:

1 $$\begin{aligned} y_{ti} \sim N\left( a_i + g_i e^{-r_i M_{ti}}, \varepsilon _i^2\right) . \end{aligned}$$

**Fig. 1:**
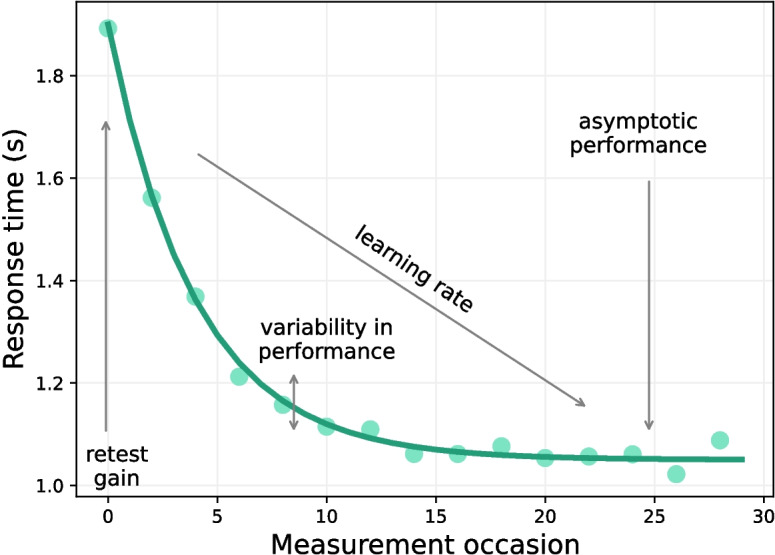
The exponential learning model. The observed day-level performance data, in terms of mean response time, are represented by dots, while model fit is illustrated by a continuous negative exponential curve. This model is governed by four parameters: gain and learning rate, which control the height and the steepness/slope of the exponential curve, respectively; variability in performance, which captures the dispersion of measurements around the fitted model; and asymptotic performance, which quantifies the position of the curve’s asymptote

On the left-hand side, we have our data $y_{ti}$, which will be response times coming from repeated assessments with a cognitive task, from participant *i*, on occasions *t*. On the right-hand side, we define how these data were generated by parameterizing an assumed theoretical process. In this particular case, (1) $a_i$ captures person *i*’s asymptotic or peak performance, (2) $g_i$ quantifies person *i*’s gain in performance, (3) $r_i$ captures person *i*’s learning rate across measurements (with $M_{ti}$ denoting person *i*’s measurement time across *t* occasions), and finally, (4) $\varepsilon _i$ is the standard deviation of the time-and-person-specific error (noise) term, to capture person *i*’s intra-individual variability (i.e., performance inconsistency, see, e.g., Dzierzewski et al., 2013). These four cognitive markers are illustrated graphically in Fig. 1. For ease of exposition, and also to potentially abstract away the generative model itself, we will often collect all process model parameters of person *i* in a vector of latent variables, ${\varvec{\Lambda }}_{i}$.

### Structural Model to Combine Observable and Unobservable Predictors

A typical next step would be to regress the latent cognitive parameters on a set of covariates or predictors. For example, considering the asymptote parameter $a_i$, which represents a person *i*’s peak performance (disentangled from practice effects), researchers might want to know if individual differences in asymptotic performance on a cognitive domain are meaningfully related to other person-level characteristics, such as sex, age, ethnicity, or some clinically meaningful outcome like MCI, genetically inherited AD status, or suicidal ideation. In this case, one might choose to regress the person-specific estimates of asymptote ($a_i$) on those manifest variables, such as $a_i \sim N(\beta _{a} \textbf{x}_i, \sigma _{a}^2)$, where $\beta _{a}$ is a set of regression weights corresponding to person-level covariates $\textbf{x}_i$, and $\sigma _{a}^2$ captures residual variation. This step is useful for establishing that individual differences in latent features are meaningfully related to person characteristics, such as asymptotic performance in our case would typically be related to age or mild cognitive impairment status as a characteristic and not as an outcome.

In applied healthcare settings, a clinician might want to use the results on the latent cognitive markers extracted from the repeated assessments differently: they want to predict the probability of a clinical outcome, such as MCI status. This could be useful, for example, if they have access to remotely collected response time data but lack the resources or access needed to bring an individual back to the clinic for comprehensive neuropsychological testing for establishing MCI status. Alternatively, such prediction could be part of some continuous monitoring for dementia risk that is based on remote ambulatory testing on people’s smartphones. To be able to offer these inferences, we will establish predictive links directly between our latent cognitive markers and MCI status, while also appropriately accounting for demographic characteristics. That is, while in many empirical studies, MCI is used on the right-hand side of predictive equations—predicting sources of individual differences in the latent cognitive features on the left-hand side—here, we want to treat it as an outcome.

To establish the usefulness of the cognitive features for prediction, our analysis should involve a structural model making this prediction. Let us denote the manifest covariates of participant *i* with the vector $\textbf{x}_i$, their latent features with the vector ${\varvec{\Lambda }}_i$, and call their key, clinically meaningful outcome $z_i$ (i.e., something interesting to detect, e.g., MCI status). We can then write the structural model for a binary outcome variable $z_i$ as

$$\begin{aligned} {\left\{ \begin{array}{ll} \pi _i = \text {logistic}\left( \beta _0 + \mathbf{{B}}_{\lambda }{\varvec{\Lambda }}_{i} + \mathbf{{B}}_\mathbf{{x}} \mathbf{{x}}_{i}\right) \\ z_{i} \sim \text {Bernoulli}\left( \pi _i\right) , \end{array}\right. } \end{aligned}$$

in which $\mathbf{{B}}_{\lambda }$ and $\mathbf{{B}}_\mathbf{{x}}$ are the vectors of regression weights that apply to the latent and manifest predictor vectors, respectively.

For our running example, we may try to predict MCI status from our latent cognitive markers while accounting for manifest demographic variables such as age, sex, education level, and racial and ethnic differences. We would then choose

2 $$\begin{aligned} {\left\{ \begin{array}{ll} \mathbf{{B}}_{\lambda }{\varvec{\Lambda }}_{i} = \beta _{a}{a_i} + \beta _{g}{g_i} + \beta _{r}{r_i} + \beta _{\varepsilon }{\varepsilon _i}\\ \mathbf{{B}}_\mathbf{{x}} \mathbf{{x}}_{i} = \beta _{age}\text {Age}_i + \beta _{sex}\text {Sex}_i + \beta _{edu}\text {Edu}_i + \beta _{rac}\text {Rac}_i + \beta _{eth}\text {Eth}_i\\ \pi _i = \text {logistic}\left( \beta _0 + \mathbf{{B}}_{\lambda }{\varvec{\Lambda }}_{i} + \mathbf{{B}}_\mathbf{{x}} \mathbf{{x}}_{i}\right) \\ \text {MCI}_{i} \sim \text {Bernoulli}\left( \pi _i\right) , \end{array}\right. } \end{aligned}$$

where $\text {MCI}_{i}$ is person *i*’s MCI status, being predicted by a logistically transformed linear combination of predictors on the right-hand side, namely $\beta _0$ denoting the intercept, coefficients $\beta _{a}$, $\beta _{g}$, $\beta _{r}$, and $\beta _{\varepsilon }$ capturing the effect of our latent predictors $a_i$ (asymptote), $g_i$ (gain), $r_i$ (learning rate) and $\varepsilon _i$ (intra-individual variability), respectively, and coefficients $\beta _{age}$ (Age), $\beta _{sex}$ (Sex), $\beta _{edu}$ (years of education, abbreviated to “Edu”), $\beta _{rac}$ (race, abbreviated to “Rac”), and $\beta _{eth}$ (ethnicity, abbreviated to “Eth”) quantifying the effect of their associated manifest predictors. The deterministic parameter $\pi _i$ will be referred to as the “MCI risk,” as it is the model-inferred probability that participant *i* has MCI.

### Inference with the Joint Bayesian Multilevel Model

**Fig. 2:**
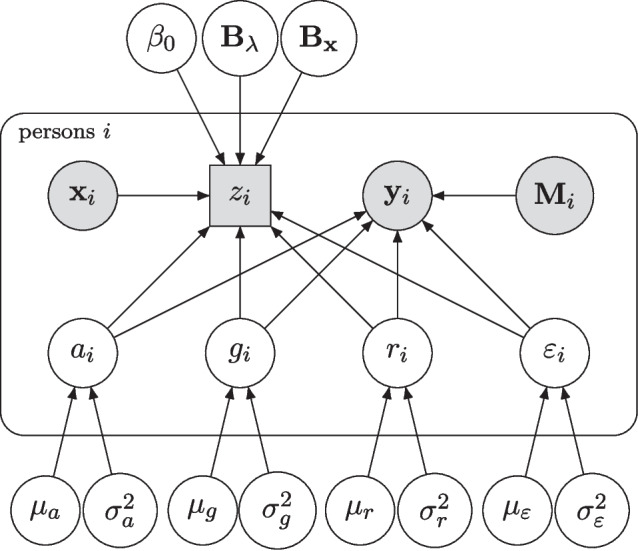
A graphical model representation of our predictive model. In the graphical model formalism, unshaded nodes indicate parameters, shaded nodes indicate observed data, and square nodes indicate discrete values. Nodes that receive an arrow are partly determined by the node where the arrow originates. Plates indicate repetitions of their contents. The figure shows the four parameters of the model of practice: $a_i$, $g_i$, $r_i$, and $\varepsilon _i$. Each of these latent parameters is person-specific and is used to predict the behavioral data $\mathbf{{y}}_{i}$, which is the vector of response times provided by person *i*. $\mathbf{{y}}_{i}$ is additionally informed by the data node $\mathbf{{M}}_{i}$ (a vector of measurement times, or more generally information about the data collection design). The latent parameters are then used again to predict $z_{i}$, the mild cognitive impairment diagnosis. In that prediction, there is an intercept $\beta _0$, the latent variables have regression weights contained in $\mathbf{{B}}_{\varvec{\Lambda }}$, and additional manifest variables $\mathbf{{x}}_{i}$ have regression weights contained in $\mathbf{{B}}_\mathbf{{x}}$

The latent cognitive markers in the process model are estimated with uncertainty. We join the two component models in a multilevel Bayesian framework so that the uncertainty in prediction from different error sources is propagated in a statistically sound manner (Boehm et al., 2018; Etz & Vandekerckhove, 2018; Wagenmakers et al., 2018). This approach allows for simultaneous estimation of cognitive markers, all regression coefficients, and variance components. The model is illustrated as a directed acyclic graph in Fig. 2 (see, e.g., Lee and Wagenmakers, 2014, for more on the graphical model formalism). We can see there that the latent parameters simultaneously inform the cognitive performance data and the MCI status data. Multiple arrows emanating from each latent predictor are critical to the POP model, as the parameters must be constrained by the behavioral data in order to be identifiable and usable as predictors in the structural component model.

For our running example, this means specifying prior and hyperprior distributions for all parameters. For the process model parameters, we pool the person-level estimates via group-(population)level distributions:

3 $$\begin{aligned} {\left\{ \begin{array}{ll} a_i \sim N(\mu _{a}, \sigma _{a}^2), \\ g_i \sim N(\mu _{g}, \sigma _{g}^2), \\ r_i \sim N(\mu _{r}, \sigma _{r}^2), \\ \varepsilon _i \sim N(\mu _{\varepsilon }, \sigma _{\varepsilon }^2). \end{array}\right. } \end{aligned}$$

For the hyperparameters, we typically choose non-informative priors that will not bias the estimation. On the means, we choose $\mu _\cdot \sim N(0,1)$, and on the standard deviations, we set $\sigma _\cdot \sim N_+(0,1)$ (the positive half-normal distribution). For the regression coefficients, we set similarly non-informative priors: $\beta _{c} \sim N(0,1)$, where the *c* subscript stands in for 0 (intercept), *a*, *g*, *r*, $\varepsilon$ (latent predictors), Age, Sex, Edu, Rac, and Eth (manifest predictors) in our current example. These prior settings assume that all covariates are standardized or dummy-coded.

The Bayesian implementation ensures that even when limited data is available (i.e., not much power to detect an effect), the resulting inferences are correct given that amount of data (but possibly with high posterior uncertainty; Wagenmakers et al., 2018). It also allows for non-binary statistical inference: Given the current data, we can easily express how much evidence we have for an effect. In our example, we might express the amount of evidence the data provide in favor of the predictive power of a particular predictor for MCI.

## Application: Predicting Mild Cognitive Impairment in the Einstein Aging Study

### The Einstein Aging Study

#### Study Design

The Einstein Aging Study (EAS) is an ongoing longitudinal research project examining risk factors for MCI and dementia. Participants of the EAS, all English-speaking, ambulatory residents of Bronx County, NY, aged 70 and above, were enlisted from local registered voting lists. The study was approved by the Albert Einstein College of Medicine Institutional Review Board, and all participants gave written informed consent.

The latest analysis includes data from 316 participants. The average age of the sample at the outset of the study was 77.54 years, with a standard deviation of 4.98 years, and 67% were female ($n = 105$ male, and $n = 211$ female). The participant pool of the study reflected a diverse mix of racial and ethnic backgrounds, with 46.2% ($n = 146$) identifying as non-Hispanic Whites, 39.9% ($n = 126$) as non-Hispanic Blacks, 9.8% ($n = 31$) as Hispanic Whites, 2.9% ($n = 9$) as Hispanic Blacks, 1.0% ($n = 3$) as Asian, and 0.3% ($n = 1$) as more than one race/ethnicity. The average educational level of the sample was 15.09 (SD = 3.55) years. Utilizing the Uniform Data System (UDS) neuropsychological assessment battery supplemented with the Free and Cued Selective Reminding Test (Katz et al., 2021) and Jak-Bondi criteria (Jak et al., 2009), 29.1% ($n = 92$) of participants were classified as having MCI at the study’s baseline.

The EAS utilizes a measurement burst design, combining frequent ecological momentary assessments with in-clinic neuropsychological tests and demographic data collection. Over a 16-day period, participants engage in six short sessions daily—each lasting no more than five minutes—using smartphones provided by the study. These sessions, conducted during usual waking hours across various daily settings, consist of cognitive tasks (“brain games”) and brief surveys on their immediate experiences, though the latter is not part of the current study’s analysis. Four out of the six daily sessions are prompted at intervals of about 3.5 h by random beeps throughout the day, while the first and last sessions are initiated by the participants themselves. In this study, numerous cognitive domains are assessed, but the current focus is on response time (RT) data from the Symbol Search task, which evaluates processing speed. The analysis focused on the daily mean response times.

#### Cognitive Assessments with the Symbol Search Task

**Fig. 3:**
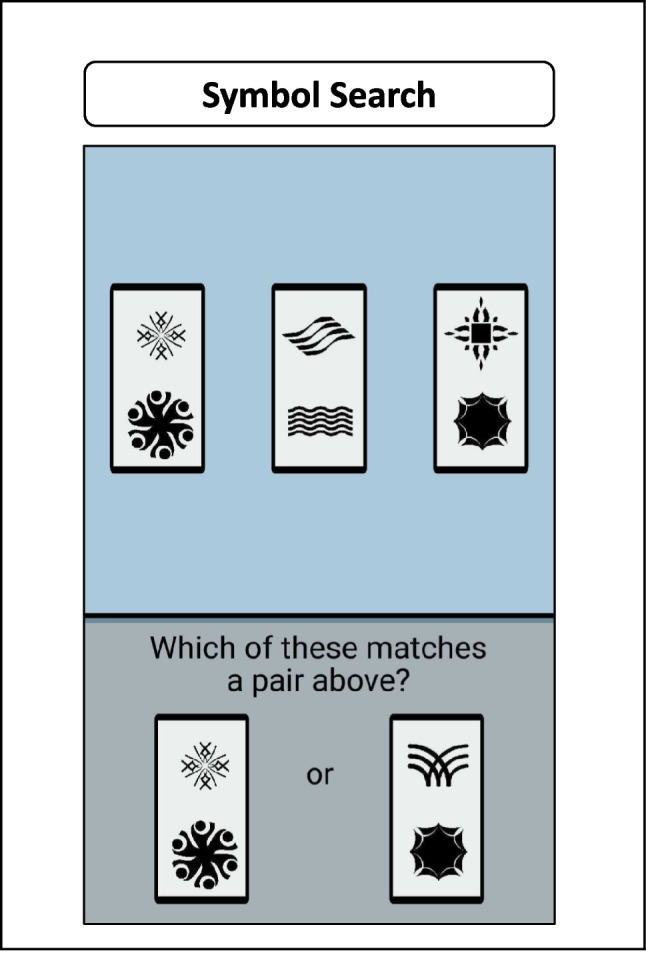
Illustration of the Symbol Search cognitive task from the Einstein Aging Study

In the current study, the Symbol Search task, depicted in Fig. 3, is used to assess processing speed. During each trial, participants were presented with three pairs of symbols at the top of the screen and two pairs at the bottom. The task required participants to quickly and accurately identify which one of the bottom pairs matched one of the top three pairs. Each session consisted of 11 trials. For analysis, we compiled daily aggregates of the reaction times (RTs) for correctly matched trials and examined them using the Bayesian Exponential Model.

#### Demographic Variables

Participant demographic data were obtained through questionnaires. For the purposes of the present analysis, we used the following demographic variables: age (expressed in years and standardized), sex (categorized as male or female based on participants’ self-declared sex, with male serving as the reference category), educational attainment (measured in total years of schooling and standardized), and ethnicity (classified as Caucasian, African American, Hispanic White, Hispanic Black, Asian, or Other). Wang et al. (2021) provide a detailed discussion of these demographic variables and their relevance for predicting MCI status.

**Table 1:** Regression coefficient estimates and their corresponding directional probabilities and estimates of the hierarchical parameters of the latent predictors with 95% credible intervals

Predictors	Mean	*P* (negative)	*P* (positive)
Intercept	$-$2.8047	1.0000	0.0000
Asymptote	0.6362	0.0027	0.9973
Learning rate	$-$1.4253	0.9923	0.0077
Intra-individual variability	0.8513	0.0671	0.9329
Gain	$-$0.0339	0.5790	0.4210
Age (standardized)	0.1929	0.0794	0.9206
Sex (1: male)	$-$0.1975	0.7606	0.2394
Years of Education (standardized)	0.1907	0.0985	0.9015
Race (1: Black)	0.4855	0.0469	0.9531
Ethnicity (1: Hispanic)	0.3031	0.2391	0.7609
Group-level summaries	Mean	PCI 2.50%	PCI 97.50%
Asymptote mean	2.9533	2.8468	3.0606
Asymptote SD	0.9053	0.8170	1.0056
Learning rate mean	0.5367	0.4686	0.6106
Learning rate SD	0.2989	0.2462	0.3604
Intra-individual variability Mean	0.7658	0.7145	0.8178
Intra-individual variability SD	0.4436	0.4011	0.4903
Gain mean	1.8067	1.6276	1.9913
Gain SD	1.1512	1.0073	1.3037

#### Mild Cognitive Impairment Status

Each participant was subjected to a comprehensive neuropsychological evaluation to determine their cognitive status. This was an in-clinic assessment and encompassed tests for memory, executive function, attention, language, and visuospatial skills (Katz et al., 2021). The criteria for MCI classification adhered to the Jak-Bondi criteria (Jak et al., 2009).

### Results

#### Implementation

We implemented the model (using Eqs. 1–3) in Stan (Carpenter et al., 2017), which can be accessed from R (R Core Team, 2022) through the rstan package (Stan Development Team, 2023). Code is available at the OSF page of the study: https://osf.io/4qpxs/. We analyzed the EAS data by running 4 parallel chains, 2500 warm-up iterations plus 2500 posterior samples per chain, for a final posterior sample size of 10,000. We did not find any problems with convergence based on the diagnostic criterion $\hat{R}$ (Gelman et al., 2013; all $\hat{R}<1.03$) and visual check of the sample chains. We checked the quality of the sampling by calculating effective sample size (the proportion of samples that can be considered as non-correlated draws in the posterior), which showed good sampling quality (above 300 for 99.8% parameters and above 1000 for 98% of the parameters, including all the key hierarchical parameters). Analysis took less than 20 min on a MacPro laptop.

#### Model Fit

We checked model fit first with a visual inspection of the correspondence between each individual’s observed data and their model-predicted learning curve (all generated plots are available on the OSF page of the project). Then, we used those data and curves to compute the proportion of variance in the data that is explained by the process model (akin to an $R^{2}$ statistic), which was .83. Both methods showed acceptable fit. However, both of these methods focused only on the fit of the process model to the observed data, and not on the prediction of MCI with the logistic regression component from Eqs. 2 (for which, see the “Predictive Accuracy” section).

#### Parameter Estimates

The top part of Table 1 shows the results from the logistic regression coefficients linking the latent features and manifest covariates to MCI status. The first column shows the name of the predictors/covariates, the second the posterior mean as a point estimate for their corresponding regression coefficient, and the last two columns display the directional probability. Instead of using a binary rule, such as excluding zero from a 95% interval, this allows for a graded approach to quantify support for effects. In this application, we will consider coefficients with directional probabilities between 91 and 95% as possibly credible effects, between 95 and 99% as likely credible effects, and over 99% strongly credible effects. The intercept is the first one of these, with its entire posterior mass in the negative range, suggesting an overall larger probability of not having MCI than having MCI in this sample, which makes sense given that only about one-third of the whole sample had MCI. From our latent cognitive markers, two showed credible links to MCI status: higher asymptotic performance (i.e., slower peak performance response time) and slower learning rate both predicted MCI. Regarding our manifest predictors, being Black tended to correspond to a higher probability for MCI. There was weak evidence that more intra-individual variability (i.e., inconsistency in performance) and older age were also predictive for MCI status. The bottom part of Table 1 additionally shows estimates of the hierarchical parameters of the latent predictors with corresponding 95% credible intervals.

#### Interpretation of Parameters

**Fig. 4:**
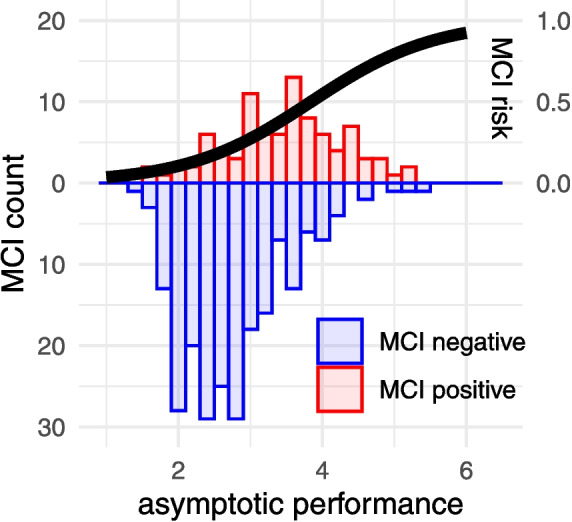
An illustration of the nonlinear predictions made by the partially observable predictor model. On the horizontal axis is the person-specific estimate of the asymptote parameter $a_i$. The downward-pointing (blue) histogram shows the number of participants in the MCI-negative group as a function of $a_i$, and the upward-pointing (red) histogram shows the corresponding number of participants in the MCI-positive group. The red histogram is somewhat to the right of the blue histogram, indicating that participants in the MCI-positive group tend to have higher asymptotes $a_i$. The thick black curve shows the MCI risk for participants with average (or baseline) values on all predictors except $a_i$. As expected, the curve is near 0 in the range of $a_i$ where most participants have negative MCI status, rises to above 0.5 at $a_i \approx 4$, but does not approach 1 anywhere in the realized range of $a_i$

Using the posterior distributions of these parameter estimates in combination with Eq. 2, we can calculate how MCI risk changes as a function of our cognitive markers. For example, if we consider a participant who has average values on everything (including latent and manifest predictors, and is male, White, and non-Hispanic for the dummies), their probability of MCI is .25. However, if this individual is 1 SD higher than the average regarding their asymptotic performance (e.g., slower reaction time), this probability goes up to .37, and if they are Black, it increases further to .49. Additionally, if this individual is also 1 SD lower in learning, then the probability increases to .59. Figure 4 further illustrates the effect of the latent variable $a_i$, which captures the asymptotic performance of a participant *i*. The two participant populations (histograms of MCI-positive and MCI-negative participant numbers) visibly separate along the horizontal axis, where the latent variable is plotted. An S-shaped curve connects $a_i$ to participant *i*’s MCI risk (denoted $\pi _i$ in Eq. 2) and illustrates the effect of $a_i$ on our MCI prediction for a participant whose other predictors are otherwise at baseline (for sex, race, and ethnicity) or at the population average (for others).

### Predictive Accuracy

The goal of our POP model approach is to decompose MCI risk into constituent components with their own psychological interpretability—a mix of manifest variables like age and sex with latent variables like asymptotic (“peak”) performance. However, the interpretability of these components comes at a cost: the added complexity of the model may harm its predictive performance (“overfitting”; Hastie et al., 2009), and we may not be willing to trade much predictive accuracy for the benefit of interpretability. For this reason, we evaluate the predictive performance of our proposed model and compare it against two more conventional models: one that uses only manifest predictors and one simplified POP model that uses behavioral data but no process model to aid in interpretability.

**Fig. 5:**
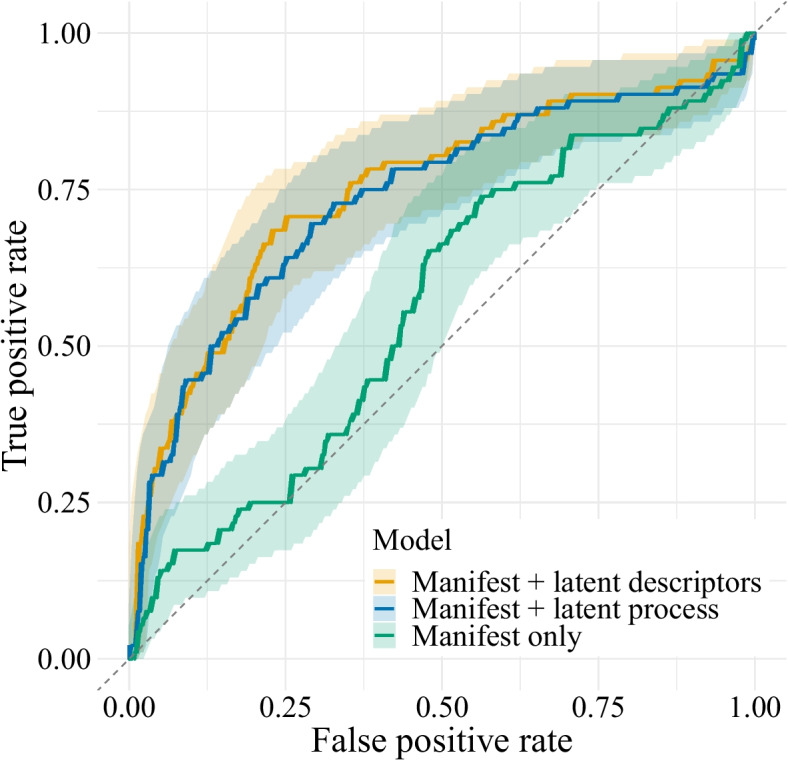
Receiver operating characteristic (ROC) curve of two partially observable predictor models, compared to a model that only uses manifest predictors. On the vertical axis is the true positive rate (correct predictions of positive MCI status) and on the horizontal axis is the false positive rate (incorrect predictions of positive MCI status). The area under the curve (AUC) is a concise summary of the predictive ability of the model. The AUC is lower for the “Manifest only” model but indistinguishable for the “Manifest + latent descriptors” and “Manifest + latent process” models. Shaded bands around the ROC curve indicate a 95% confidence interval obtained through a bootstrap procedure (Carpenter & Bithell, 2000; Robin et al., 2011)

#### Evaluation Metrics

To evaluate predictive performance, we calculated the area under the curve (AUC) of the receiver operating characteristic (ROC) curve, which summarizes the diagnostic ability of a binary classifier system as its discrimination threshold is varied (Swets, 1988). The AUC is a preferred statistic especially when dealing with imbalanced datasets in which accuracy alone may be misleading.

#### Cross-validation

We calculated the AUC via a ten-fold cross-validation procedure (Hastie et al., 2009). From the original data set ($N = 316$), we first created ten subsets. We stratified the subsets such that, like the full data set, each subset contained about 30% MCI-positive participants. We then fit the model ten times, each time holding out the MCI status of a different subset of participants. We estimated their latent process parameters from their cognitive task data and combined those with their manifest predictors to obtain a person-specific predicted MCI risk $\pi _i^\text {pred}$ for each holdout participant. We then created a ROC curve by varying the critical value of $\pi _i^\text {pred}$ by which we categorized participants as MCI negative or positive, and we computed a confidence interval around the ROC curve using a bootstrap procedure provided by R’s pROC package (Carpenter & Bithell, 2000; Robin et al., 2011).

#### Comparator Models

For comparison, we then similarly calculated ROC curves for a model using only the manifest predictors (the “Manifest only” model) and a POP model that combines manifest predictors with a simplified generative model of RT (describing RT only in terms of mean and standard deviation; the “Manifest + latent descriptors” model).

#### Results

ROC curves for the three models are shown in Fig. 5. The AUC of the “Manifest + latent process parameters” model was .7346 (95% CI, [0.6680, 0.8013]). The AUC of the “Manifest only” model was.5640 (95% CI, [0.4938, 0.6342]), with its confidence interval including .5, and the AUC of the “Manifest + latent descriptors” model was .7371 (95% CI, [0.6717, 0.8026]).

#### Discussion

The results of this cross-validation exercise led to three conclusions. First, the lower AUC for the “Manifest only” model speaks to the predictive utility of behavioral data above and beyond demographics. The AUC for this model was close to .5, suggesting that predicting MCI status based solely on demographic information yields accuracy nearly equivalent to random guessing. Second, the observation that the AUCs and ROCs for the “Manifest + latent process parameters” and “Manifest + latent descriptors” were indistinguishable assuages any concerns of overfitting by the more complex process model. Third, the AUCs of the POP models are fair but also leave room for improvement (see, e.g., the examples in Swets, 1988). Future work focusing on predictive accuracy could use POP models to factor in additional sources of evidence, possibly from a variety of tasks in a battery. However, for the current data, we conclude from this analysis that the “Manifest + latent process parameters” model improves interpretability with no concomitant reduction in predictive accuracy.

## Conclusions and Discussion

There is a growing consensus that 40% of dementia cases are due to modifiable risk factors (Lee et al., 2022). Understanding the early signs of subtle cognitive decline, for example, in preclinical ADRD, can open up new possibilities for secondary prevention and monitoring. These subtle early cognitive changes are most likely related to specific subprocesses that we need to identify. We have introduced partially observable predictor (POP) models, an approach that combines observable demographic variables with unobservable predictors derived from behavioral data. We demonstrate how this approach may be applied to test the role of cognitive process parameters for early detection. POP models let us discern which underlying psychological components are relevant for predicting clinically meaningful outcomes, offering a clearer picture on early-stage neuropsychological impairments.

### Conclusions from the Einstein Aging Study

Using the Einstein Aging Study (EAS) data set, we have demonstrated that data from repeated cognitive measures improved prediction accuracy when compared to a model with only manifest variables. Furthermore, we were able to decompose the contribution of these cognitive measures using a cognitive process model, which allowed us to compare the individual contribution of interpretable cognitive subprocesses. For the processing speed task in the EAS, the learning rate and peak performance turned out to be important for the prediction of mild cognitive impairment.

#### Limitations of the Example Data Set

Even though our predictive model pooled information from multiple sources, total prediction accuracy was lower than in some previously published studies (e.g., Oh et al., 2024; Yan et al., 2023). This was true independently of the application of the POP model (i.e., it was also true when using only the manifest predictors), leading us to believe that the MCI categorization used in the EAS data (Chang et al., 2024) was noisier than other methods (Devlin et al., 2022). Indeed, in longitudinal data with this categorization method, participants even occasionally changed status from positive back to negative over time.

#### Potential for Future Uptake

Since the response time data that we used to derive our cognitive markers was collected in ambulatory settings—specifically, via smartphones—the approach facilitates easy screening and monitoring of mild cognitive impairment risks. These measures represent naturalistic, real-time functioning. While neuropsychological evaluations are widely regarded as the definitive method for identifying cognitive deficits, they are extensive and generally need to be conducted in person, restricting their broad applicability in large-scale research and clinical studies. The EAS data were collected with a cognitive task that could easily be collected remotely, multiple times per year, representing a low barrier for entry for underrepresented populations who might not have easy access to clinicians.

We are cautiously optimistic about the broader appeal and potential for uptake of our proposed approach. Given that cognitive researchers could use POP models to incorporate many different process models—whichever is more appropriate for the behavioral data at hand—into a one-step predictive model, we anticipate interest. The key barrier to uptake is the technical challenge involved in the implementation of a Bayesian multilevel model in Stan. However, Bayesian methods and models are no longer the obscure niche skill they once were—the increase in relevant course books and tutorials (Lee & Wagenmakers, 2014; McElreath, 2020; Vandekerckhove et al., 2018; Wagenmakers et al., 2018) strongly suggests that Bayesian cognitive modeling is becoming a leading paradigm in computational cognitive science that is suitable for translation to clinical science (Huys et al., 2016; Maia & Frank, 2011). Additionally, we have made all of our data analysis code (Stan/R scripts and an associated Dockerfile) freely available via OSF. To facilitate adoption in this field even more, it would be optimal to have a user-friendly online platform that integrates data collection with the delivery of analytical results to clinicians. Although we do not currently have such a platform, building such tools has been an NIH funding priority (e.g., National Institute on Aging, 2022), and recent progress (e.g., Hakun et al., 2024) gives us confidence in these becoming a reality.

#### Future Work

Further studies that use POP models in different data sets might identify other subprocesses extracted from cognitive tasks capturing performance on a different cognitive domain, and a combination of information from multiple multi-domain tasks could lead to increased predictive accuracy, but more importantly to greater understanding that could inform targeted intervention.

Secondly, while we used concurrent MCI status in the current application, it is possible to gather data on which non-MCI participants at baseline develop MCI in the future (“incident” MCI). In future work, we will use this approach for prediction in the epidemiological sense, with a focus on predicting an outcome in the future based on information in the present.

Finally, we note that while we focused on cognitive models, the presented approach can be applied across various fields. In computational psychiatry, for instance, a comparable strategy is used to distill latent emotional dynamics, which can then inform predictive models for critical clinical outcomes like suicide risk.

## Data Availability

The data analyzed in this study is subject to the following licenses/restrictions: Interested collaborators are asked to complete a concept proposal form (details for potential project, paper, or abstract) to be reviewed and forwarded to the Einstein Aging Study Steering Committee for consideration. Requests to access these datasets should be directed to MK, MPH at mindy.katz@einsteinmed.org. For additional information on data sharing requests for the Einstein Aging Study, https://einsteinmed.edu/departments/neurology/clinical-research-program/eas/data-sharing.aspx .

## Footnotes

1 For example, we often see models of the form $\theta _p \sim N\left( \beta _0 + \beta _1\text {MCI}_p, \sigma ^2\right)$ (or nonlinear variations thereof) in which the clinical state of person *p* ($\text {MCI}_p$) is treated as known while the cognitive parameter $\theta _p$ is treated as the unknown. Realistically, both the latent parameter $\theta$ and the clinical state are unknown, and the latter must be inferred from diagnostic data.

## Code Availability

Code is available at the OSF page of the study: https://osf.io/4qpxs/.
